# Patient pathways of tuberculosis care-seeking and treatment: an individual-level analysis of National Health Insurance data in Taiwan

**DOI:** 10.1136/bmjgh-2019-002187

**Published:** 2020-06-21

**Authors:** Chu-Chang Ku, Chien-Chou Chen, Simon Dixon, Hsien Ho Lin, Peter J Dodd

**Affiliations:** 1 School of Health and Related Research, The University of Sheffield, Sheffield, UK; 2 Center for Applied Artificial Intelligence Research, Soochow University, Taipei, Taiwan; 3 Institute of Epidemiology and Preventive Medicine, National Taiwan University, Taipei, Taiwan

**Keywords:** tuberculosis, health economics, health systems evaluation, public health, cohort study

## Abstract

**Introduction:**

Patients with tuberculosis (TB) often experience difficulties in accessing diagnosis and treatment. Patient pathway analysis identifies mismatches between TB patient care-seeking patterns and service coverage, but to date, studies have only employed cross-sectional aggregate data.

**Methods:**

We developed an algorithmic approach to analyse and interpret patient-level routine data on healthcare use and to construct patients’ pathways from initial care-seeking to treatment outcome. We applied this to patients with TB in a simple random sample of one million patients’ records in the Taiwan National Health Insurance database. We analysed heterogeneity in pathway patterns, delays, service coverage and patient flows between different health system levels.

**Results:**

We constructed 7255 pathways for 6258 patients. Patients most commonly initially sought care at the primary clinic level, where the capacity for diagnosing TB patients was 12%, before eventually initiating treatment at higher levels. Patient pathways are extremely heterogeneous prior to diagnosis, with the 10% most complex pathways accounting for 48% of all clinical encounters, and 55% of those pathways yet to initiate treatment after a year. Extended consideration of alternative diagnoses was more common for patients aged 65 years or older and for patients with chronic lung disease.

**Conclusion:**

Our study demonstrates that longitudinal analysis of routine individual-level healthcare data can be used to generate a detailed picture of TB care-seeking pathways. This allows an understanding of several temporal aspects of care pathways, including lead times to care and the variability in patient pathways.

**Key questions**

**What is already known?**

- Care-seeking cascades for people with tuberculosis (TB) disease have mainly been investigated in low-income and middle-income countries and relied on cross-sectional data that do not include details of patient interactions with health systems and their timing.
- Patient pathway analysis methods have been introduced to identify misalignment of TB service provision with patient demand using cross-sectional data from disparate sources.
- Under universal health coverage, routine health insurance data in Taiwan capture detailed individual records, but events require interpretation to reconstruct individual patient pathways.

**What are the new findings?**

- To analyse these data, we developed a new individual patient pathway analysis (IPPA) framework and explored the temporal patterns, complexity and heterogeneity of TB patient pathways.
- Applying IPPA to the Taiwan data from 2000 to 2010, we found huge heterogeneity in pathways driven by the interruption of evaluation and re-evaluation: 10% of the pathways had delays of over 200 days between initial care-seeking and treatment initiation, while the median delay was 41 days.

**What do the new findings imply?**

- Routinely collected data can help understand the care-seeking of patients with TB.
- Understanding the causes of interrupted evaluation should be a focus of further investigation in Taiwan. Lower attention to TB as a cause of illness with declining burden may play a role in this setting.

## Introduction

Tuberculosis (TB) is a curable infectious disease but has become the world’s leading infectious killer with unacceptably slow progress towards meeting global targets laid out in the End TB strategy.^1^ These targets include the elimination of catastrophic costs due to TB,^2^ in recognition of the financial impact TB illness and care-seeking imposes on households.^3^ One of the key gaps in TB control is the ‘missing millions’: 4 in 10 people developing TB globally are either not diagnosed and treated, or are not notified to national tuberculosis programmes (NTPs).^1^ Understanding the health system pathways of patients who are seeking care for TB is key in identifying low-hanging fruit for TB control: programmatic shortcomings that mean patients are either missed, not notified or incur ruinous expense in their efforts to obtain care.

Cascade of care analyses for TB have addressed the drop-offs at different stages from those needing treatment through to those completing treatment for active tuberculosis^4^ and for latent TB infection.^5^ This work has recently been extended with the introduction of patient pathway analysis (PPA) for TB,^6^ which provides a standardised analysis approach for drawing together data from different sources to identify misalignment between capacity for TB diagnosis and treatment, and patients’ preferences in seeking care.^7^ Initial work was undertaken in five countries^8–12^ and notably found low coverage of diagnostic capacity for TB at facilities where patients typically first present.^13^ This work has argued that more needs to be done to ‘meet the patients where they are’,^14^ in line with the patient-centred care pillar of the End TB strategy.

However, work has so far relied on cross-sectional and aggregate data, meaning it has not been able to track the pathways of individuals as they interact with the health system. This makes it hard to investigate the key issue of delays prior to diagnosis. It also means it is not possible to understand patterns of referral between levels of facilities, the true complexity and heterogeneity of patient pathways, or the factors that influence these. Going beyond capacity alignment requires routine individual-level data with high coverage. Any data that include individuals’ interactions with the health system before a diagnosis of TB require a methodology to interpret these events as related to TB care-seeking in light of their characteristics and timing. In this study, we develop a generalisable methodology for individual PPA that makes use of health insurance data and apply it to Taiwan to construct and analyse the care pathways of patients who ultimately were diagnosed and treated for TB.

## Methods

### Setting

TB incidence in Taiwan has been declining: from 64 cases per 100 000 in 2007 to 38 per 100 000 in 2018. Among the incident cases in 2018, less than 1.3% of cases were MDR-TB and around 4.9% of cases were extrapulmonary TB.^15^ Since 2008, more than 50% of TB cases were aged 65 years or above, and this proportion is growing.^16^ Taiwan is a high-income country and its healthcare system is fully integrated with the National Health Insurance (NHI) programme. The NHI is compulsory (over 98% enrolment in 2017) and covers most essential healthcare services in both public and private sectors. The National Health Insurance Research Database (NHIRD) comprises records of all healthcare claims (eg, diagnostics and medications) funded by the NHI. For TB, the NTP covers TB diagnosis copayments and treatment and provides social support for essential household expenditure.^17^

### Data

A simple random sample of one million individuals from the NHIRD between 1996 and 2000 was linked to data beyond this period on individual characteristics (age and sex), healthcare records and healthcare facilities visited.^18^ The main reason for clinical visits and clinicians’ diagnoses on prescription are recorded in the NHIRD using the International Classification of Diseases, Ninth Revision, Clinical Modification (ICD-9-CM).

The NHIRD was provided by the NHI Administration, Ministry of Health and Welfare and managed by National Health Research Institutes (registered number 100290). The interpretation and conclusions contained herein do not represent those of the NHI Administration, Ministry of Health and Welfare or National Health Research Institutes.

### Construction of individual patient pathways

**Figure 1:**
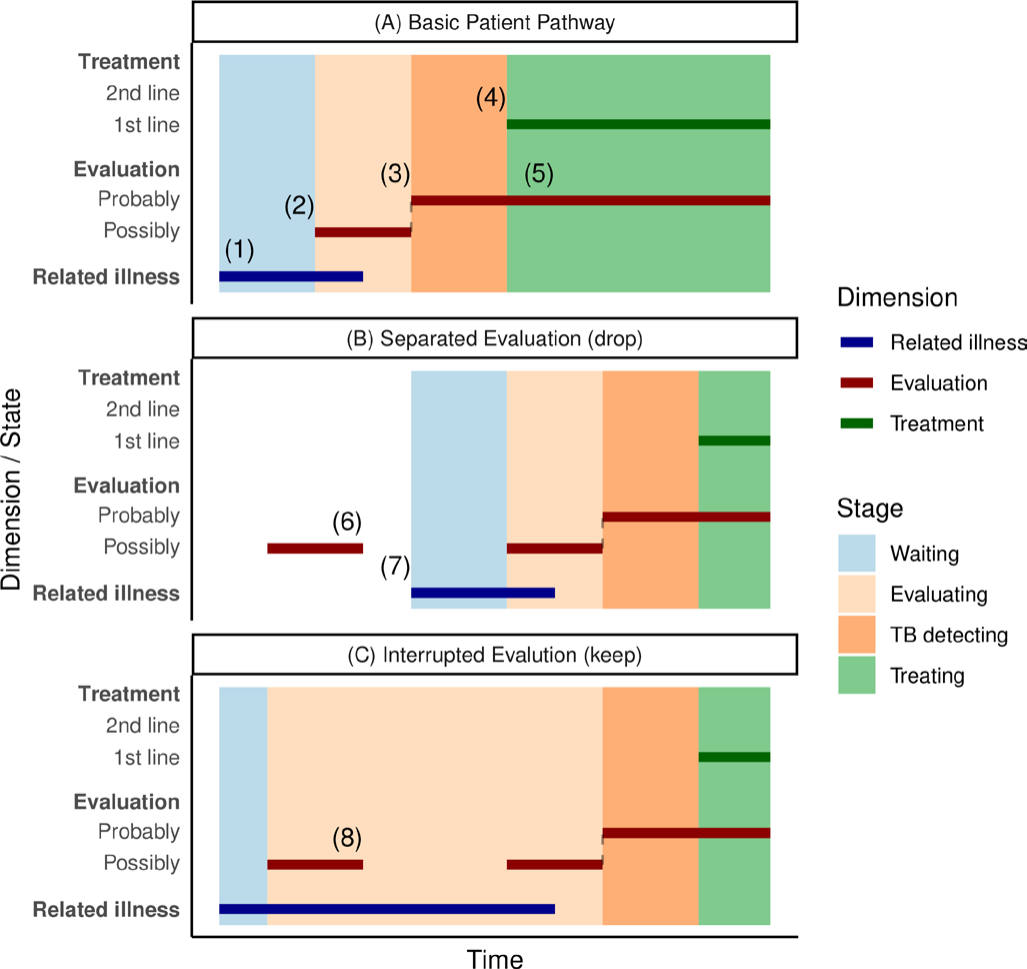
Patient pathway construction bars show patient states in three domains where non-zero. Background colours show the stage label within a pathway. (A) Basic patient pathway: (1) corresponds to initial care-seeking; (2) and (3) represent escalating clinical consideration of TB; (4) is treatment initiation; and (5) represents diagnostic evaluation while on anti-TB treatment. (B) Separated evaluation series. Considering there is no count in related illness dimension, the evaluation (6) would be dropped as separated by longer than 60 days, and the pathway would begin at (7). (C) Interrupted evaluation. With evidence of clinician consideration of a related illness, the evaluation at (8) is included in the pathway. TB, tuberculosis.

In constructing individual patient pathways, we sampled individual patients who met previously validated criteria for TB diagnosis between 2003 and 2010.^19^ We assumed their care-seeking began within 3 years prior to diagnosis and thus used their insurance records starting from 2000 in the following three-step analysis. First, we introduced a dynamic three-dimensional patient state with dimensions related to evaluation, treatment and clinician consideration of related illnesses (all with default values of zero). Second, this state was used to parse sets of records into separate episodes of TB-related care (periods with ≥1 state non-zero; see figure 1). Third, the state values were used to interpret and label the stages of each episode, resulting in a pathway. The Taiwan Centers for Disease Control (CDC) guidelines,^20^ WHO guidelines^21^ and expert opinion on current clinical practice were used to define the algorithm for state transitions and stage labelling (see online supplementary appendix 1). In the rest of this section, we describe this approach in more detail.

10.1136/bmjgh-2019-002187.supp1 Supplementary data

The evaluation dimension represents the use of diagnostic procedures relevant to differential diagnosis of TB. Evaluation tools were ranked as probably for TB and possibly for TB according to how specific their use was to TB. Evaluation possibly for TB was triggered by various tools for examining the respiratory system and antibiotics for pneumonia. Evaluation probably for TB was triggered by the use of tools, including chest CT scan, chest X-ray, acid-fast bacteria culture, *Mycobacterium tuberculosis* culture and tuberculin skin tests (see online supplementary appendix 1 for detailed definitions).

The treatment dimension represents the use of anti-TB drugs. In accordance with Taiwan CDC guidelines, anti-TB treatment requires more than two types of anti-TB drugs prescribed continuously for at least 4 weeks. Treatment was either first-line or second-line according to the drugs prescribed (see online supplementary appendix 1). We did not consider multidrug-resistant (MDR)-TB separately.

The related illness dimension captures potential clinician consideration of alternative diagnoses. Increased values were triggered by features, including ICD-9-CM codes corresponding to acute respiratory infections, chronic lung diseases and non-tuberculous mycobacterial infections.

Each state’s value could return to zero after a ‘time-out’ period if no relevant trigger records occurred. A patient’s treatment state also returned to zero after treatment completion. The choice of time out sets a memory timescale beyond which records no longer influence a patient’s state. The default time out was 60 days, and sensitivity analyses used 30, 90 and 120 days.

Care-seeking episodes were defined as collections of records including anti-TB treatment (excluding exploratory treatment), during which a patient’s state was continuously above zero in at least one dimension, separated by periods with all dimensions equal to zero (see figure 1). Initial care-seeking is defined as the start of a care-seeking episode. Episodes could include records related to care-seeking prior to TB diagnosis. Without evidence that alternative diagnoses were being actively considered (ie, related illness state and evaluation state both return to zero), prior evaluations are dropped from an episode (figure 1B). If there was evidence of ongoing consideration of alternative diagnoses to TB (non-zero-related illness state), diagnostic procedures relevant to TB would be included in the episode even if the evaluation state returned to zero in the interim (figure 1C); this is termed an ‘interrupted evaluation’.

To complete pathway construction, time within parsed episodes was labelled as one of four main stages according to the values of the state (figure 1, background colours): waiting stage (before evaluation), evaluating stage (under evaluation for possible TB), TB detecting stage (under evaluation for probable TB) and treating stage (on TB treatment).

### Analyses of individual patient pathways

We calculated descriptive statistics including the number of pathways, in total, and by gender and age.

Individual patient pathways were analysed to understand the coverage of services at different levels of the health system and referral patterns between them. We extracted the list of hospitals with captured events from the patient pathways. Hospitals were classified into one of four levels following a standard classification (A for primary care/general practice, B for regional hospitals with inpatient capacity, C for larger district hospitals and D for large hospitals with a range of specialists). We then calculated the ‘coverage’ of each service at a given level of facilities (the proportion of facilities of the given level where the service was available), as well as the patient ‘access’ to these services (proportions of levels of hospitals where patients initially sought care). We summarised these metrics with a modified version of the PPA visualisation introduced by Hanson *et al*.^7^ We also visualised the flow of patients between health service levels by pathway stage. We computed the distribution of delays from initial care-seeking to first reaching facility with anti-TB treatment capacity, to first reaching the facility where ultimately treated and to treatment initiation. System delay refers to the period between initial care-seeking to treatment initiation.

The heterogeneity in patient pathway complexity and time spent in different stages was visualised by sequence frequency plots, separately for subpathways leading up to and following treatment. To summarise time taken to reach a given stage of care, we also visualised the number of pathways reaching a given substage by time after initial care-seeking. We calculated the proportions of all clinical encounters among the pathways with the 10% least common sequence patterns; of these pathways for which treatment started over 1 year after initial care-seeking and of all pathways experiencing interrupted evaluation. Finally, we analysed risk factors, including age, sex, area, comorbidity and hospitals at initial care-seeking for interrupted evaluation using logistic regression.

The pathway extraction was performed in Python V.3.6; statistical analysis and visualisation were performed in R V.3.5.1 and ggplot2. See source code (https://github.com/PatientPathwayAnalysis/IPPA-py), online supplementary appendices 2 and 4 for implementation detail, links in online supplementary appendix 6 for online documents and related code repositories.

10.1136/bmjgh-2019-002187.supp2 Supplementary data

10.1136/bmjgh-2019-002187.supp3 Supplementary data

10.1136/bmjgh-2019-002187.supp4 Supplementary data

## Results

A total of 6258 patients met our criteria for TB between 2003 and 2010. Our algorithm generated 7255 distinct pathways for these patients: 88% of patients had only one pathway. The use of longer time outs resulted in fewer distinct pathways: 7528 for 30 days, 6963 for 90 days and 6831 for 120 days (see online supplementary appendix 3, sensitivity analysis). Men contributed 70% of patient pathways; 50.5%, 49% and 0.5% of pathways were from those aged over 65, between 15 and 64, and below 14 years, respectively. Around 16% of patient pathways involved those with chronic lung conditions. Lastly, over 50% of patient pathways occurred in the two major cities: Taipei City and Kaohsiung City (see the first column of table 1).

10.1136/bmjgh-2019-002187.supp5 Supplementary data

**Table 1:** Logistic regression analysis of risk factors for interrupted evaluation

Factors	Number	IE (%)	Crude OR (95% CI)	Adjusted OR (95% CI)	P value (Wald's test)	P value (LR test)
All	7255	1125 (16)				
Age (years)						<0.001
0-14	36	4 (11)	1.18 (0.41 to 3.34)	0.97 (0.33 to 2.8)	0.952	
15-64	3558	342 (10)	Reference			
65+	3661	779 (21)	2.54 (2.22 to 2.91)	2.42 (2.1 to 2.8)	<0.001	
Sex						0.817
Female	2158	315 (15)	Reference			
Male	5097	810 (16)	1.11 (0.96 to 1.27)	0.98 (0.85 to 1.14)	0.817	
Area						<0.001
Centre	1332	234 (18)	Reference			
East	419	76 (18)	1.04 (0.78 to 1.38)	1.12 (0.83 to 1.51)	0.458	
Kaohsiung City	1507	267 (18)	1.01 (0.83 to 1.23)	1.09 (0.89 to 1.33)	0.419	
North	740	98 (13)	0.72 (0.55 to 0.92)	0.77 (0.59 to 1)	0.054	
South	1087	179 (16)	0.93 (0.75 to 1.15)	0.95 (0.76 to 1.19)	0.672	
Taipei City	2170	271 (12)	0.67 (0.55 to 0.81)	0.75 (0.62 to 0.92)	0.005	
Comorbidity						
CLD	1192	277 (23)	1.86 (1.6 to 2.17)	1.91 (1.62 to 2.24)	<0.001	<0.001
DM	1191	136 (11)	0.66 (0.55 to 0.8)	0.64 (0.53 to 0.78)	<0.001	<0.001
HIV	30	4 (13)	0.84 (0.29 to 2.4)	1.52 (0.51 to 4.49)	0.451	0.472
Initial level						<0.001
A	3104	539 (17)	Reference			
B	1275	203 (16)	0.9 (0.76 to 1.08)	0.78 (0.65 to 0.93)	0.007	
C	1644	210 (13)	0.7 (0.59 to 0.83)	0.68 (0.57 to 0.82)	<0.001	
D	1232	173 (14)	0.78 (0.65 to 0.94)	0.75 (0.62 to 0.92)	0.005	
Pathway overlaps 2003	2110	493 (23)	2.18 (1.91 to 2.48)	2.32 (2.02 to 2.66)	<0.001	<0.001

Healthcare facility levels: A, for primary care/ general practice; B, for regional hospitals with inpatient capacity; C, for larger district hospitals; D, for large hospitals with a range of specialists.

Comorbidities and pathway overlaps 2003 are binary variables, applying 'none' as the reference groups.

Crude and adjusted ORs were calculated from the results of univariate and multivariate logistic regressions, respectively.

CLD, chronic lung disease; DM, diabetes mellitus; IE, interrupted evaluation; LR, likelihood ratio.

**Figure 2:**
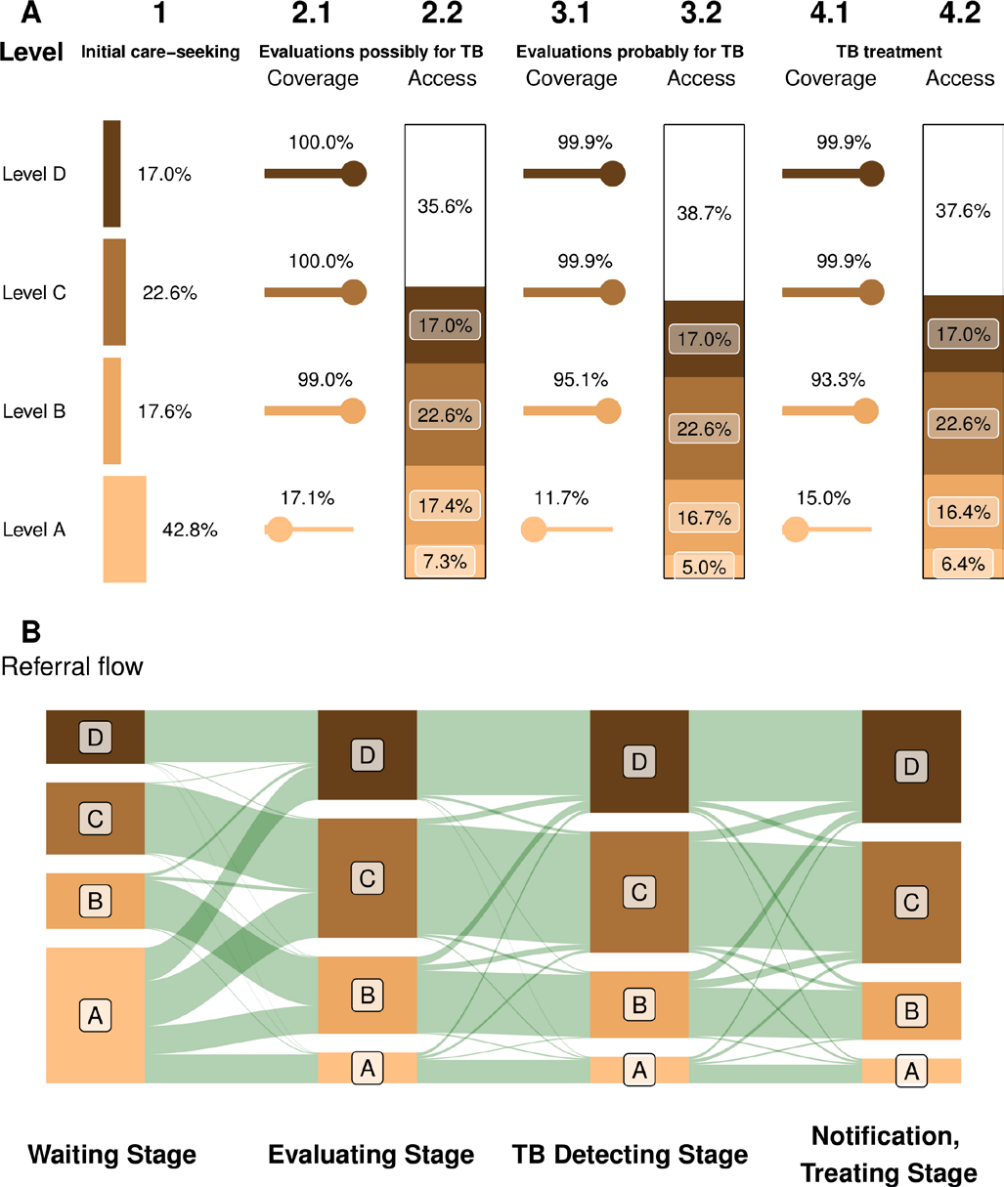
Alignment between the capacity for TB diagnosis and treatment, and patients’ preferences in seeking care hospital levels: A, for primary care/general practice; B, for regional hospitals with inpatient capacity; C, for larger district hospitals; D, for large hospitals with a range of specialists. (A) Coverage of and access to TB services at different levels of Taiwan’s health system modified. This panel is a modified version of the visualisation introduced by Hanson *et al*.^7^ Coverage is the proportion of facilities at that level offering a service; access is the product of coverage and the fraction of patients seeking care at that level. (B) Patient flows between levels at each stage, including clinician referral and self-referral (vertical heights of bands are proportional to numbers). TB, tuberculosis.

Coverage and access by level of the health system are shown in figure 2A. Initial care-seeking was most common at level A, but further evaluations and treatment rarely occurred at this level; only a minority of these facilities (around 15%) had the capacity for TB diagnosis and treatment, as shown in the bottom row of figure 2A. Coverage at facility levels higher than A exceeded 93%. Figure 2B shows the associated flow of patients between levels of the health system, with an upwards flow across all stages. Around 50% of patient pathways began at level A or B (36% where TB treatment was unavailable), but over 74% of pathways initiated treatment at level C or D.

**Figure 3:**
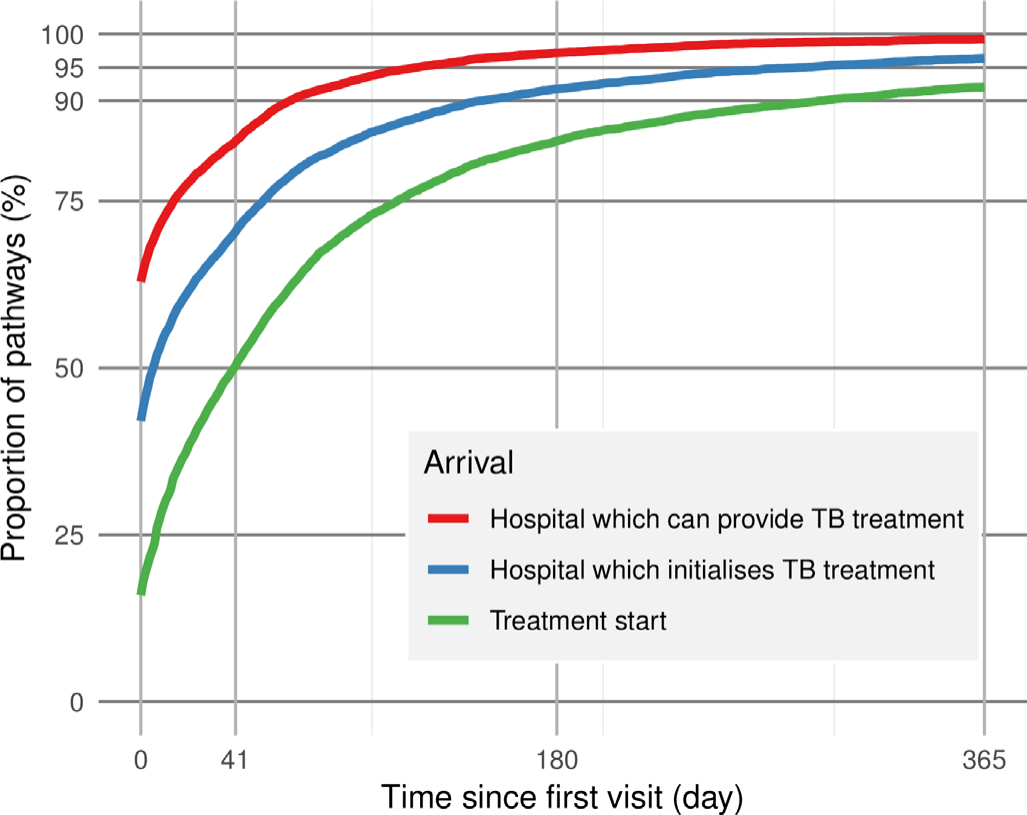
Delays from initial care-seeking; the curves indicate the proportion of pathways by time that have reached hospitals providing TB treatment (red); reached the hospital, which ultimately initiates their TB treatment (blue); and started treatment (green). The vertical line on day 41 denotes the median system delay. TB, tuberculosis.

Delays from initial care-seeking to a facility offering TB treatment, the facility ultimately providing treatment and treatment initiation are shown in figure 3. Sixteen per cent of pathways initiated treatment at their initial visit. By day 60, TB treatment was available for 90% of the pathways and 80% had arrived at the facilities ultimately providing their treatments. By day 180, 84% of pathways had started treatment. The median system delay (from initial care-seeking to treatment initiation) was 41 days.

**Figure 4:**
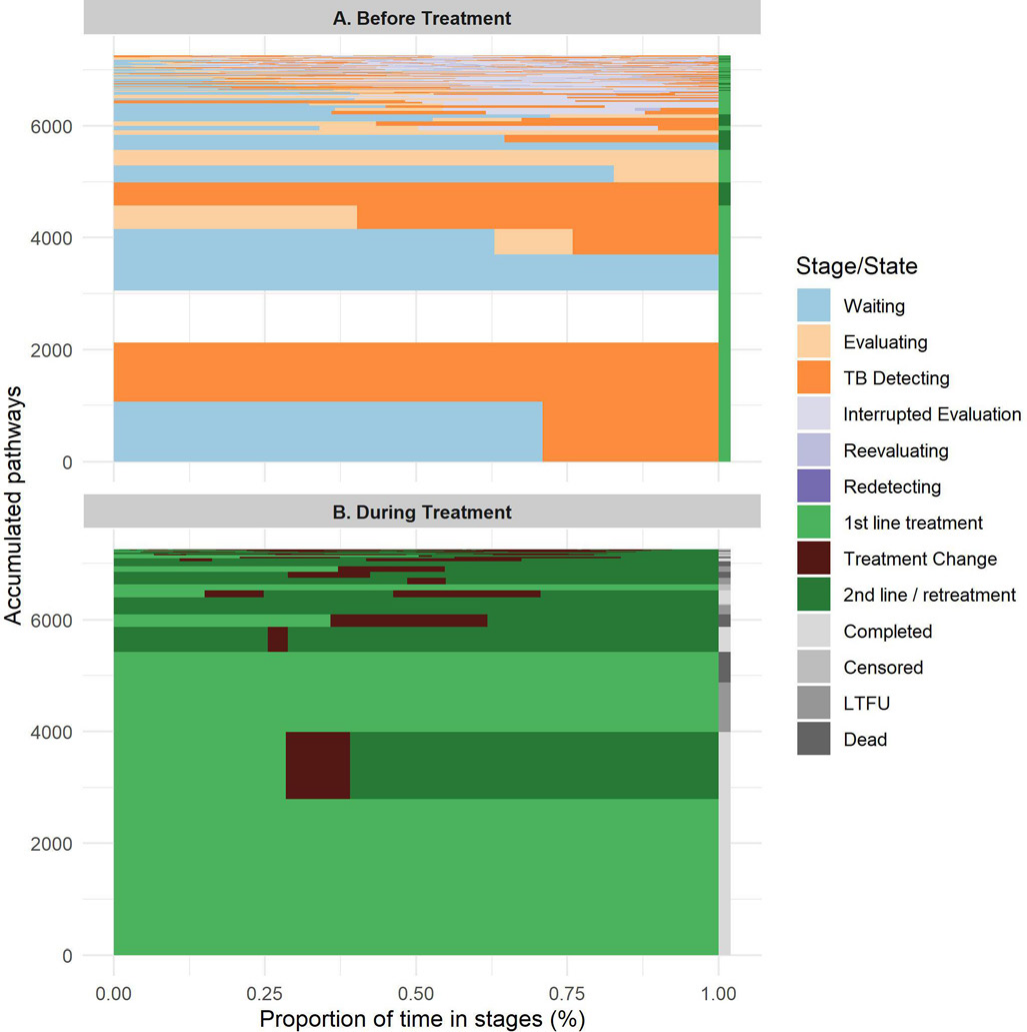
Cumulative pathway typology before and after treatment initiation. The aggregated number of pathways with a given sequence of stages (Y axis) versus the proportion of their time in each stage (X axis). (A) Sequence patterns before treatment initiation. (B) Sequence patterns from treatment initiation to treatment outcome. Completed: treatment period longer than 180 days; censored: end with the end of data time frame. See article text and online supplementary appendix 5 for stage definitions. LTFU, lost to follow-up, incomplete treatment for no reason; TB, tuberculosis.

The typology of pathways before and after treatment start is shown in figure 4. Before treatment starts, simpler pathways were most common. Second-line anti-TB treatments (including fluoroquinolones) were used in 34% of pathways. However, the most complex pathways (ie, pathways with the rarest 10% of sequence types; see figure 4) exhibited intricate patterns of evaluation interruptions and re-evaluations; 100% of these pathways included interrupted evaluation.

10.1136/bmjgh-2019-002187.supp6 Supplementary data

**Figure 5:**
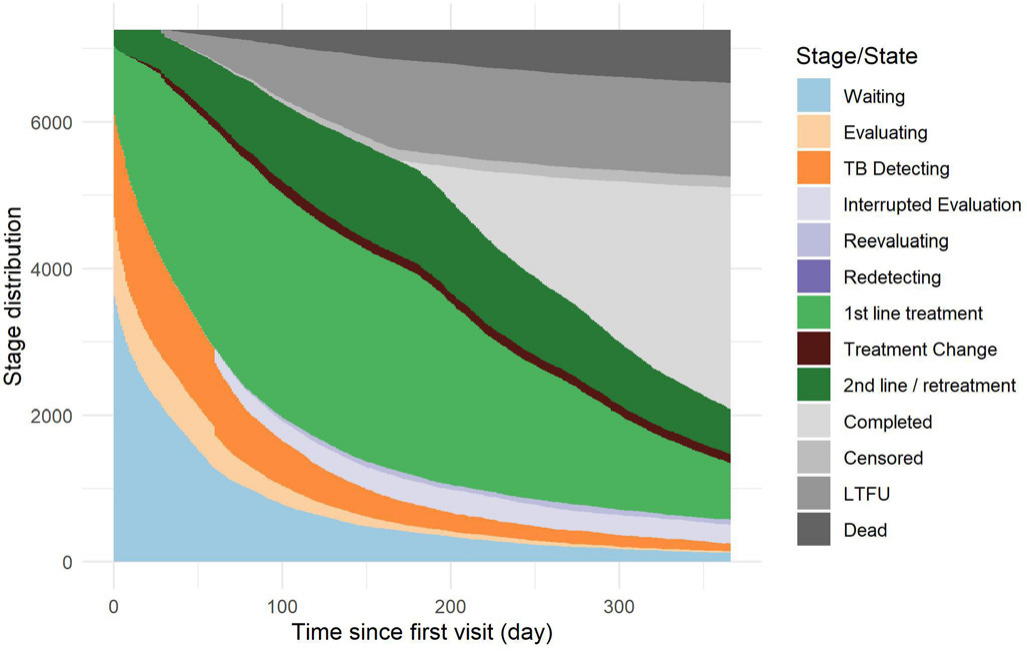
Time from initial care-seeking to given care stage completed: treatment period longer than 180 days. Censored: end with the end of data time frame. See article text and online supplementary appendix 5 for stage definitions. LTFU, lost to follow-up, incomplete treatment for no reason; TB, tuberculosis.

The pattern of delays from initial care-seeking to later stages of care is shown in figure 5, truncated at 1 year. The long tail of 7% of pathways yet to start treatment a year after initial care-seeking is clearly visible. One year after initial care-seeking, 21% of pathways were still on TB treatment (40% of these on second-line treatment); 41% had completed treatment; 18% were lost to follow-up; and 10% of patients had died. Fifty-five per cent of the 10% of most complex pathways were yet to initiate treatment a year after initial care-seeking.

Interrupted evaluation occurred in 16% of all pathways, but these pathways included 48% of all visits/records and comprised a majority of the most complex and delayed pathways (see previous discussion). The median delay from initial care-seeking to treatment initiation in pathways with interrupted evaluation was 313 days (interquartile interval 164–607 days). Table 1 shows that pathways for patients aged 65 years or older (OR 2.4, 95% CI 2.1 to 2.8) compared with patients aged 15–64 years, those with chronic lung disease (OR 1.9, 95% CI 1.6 to 2.2), and pathways which included 2003 (the year of the severe acute respiratory syndrome (SARS) epidemic) (OR 2.3 95% CI 2.0 to 2.7) were significantly more likely to experience interrupted evaluation. Pathways for patients with diabetes mellitus (DM) and who sought care at hospitals higher than level A were significantly less likely to include interrupted evaluation (OR 0.64, 95% CI 0.53 to 0.78).

## Discussion

In this study, we constructed individual patient pathways of TB care-seeking, diagnosis and treatment using routine NHI data in Taiwan. To do this, we developed a generalisable method to algorithmically interpret insurance claim records in light of the context of previous and future events on the pathway. This approach was necessary in order to determine whether healthcare use prior to eventual diagnosis was in fact related to a given TB episode. Even in this well-resourced setting, there is low apparent coverage of TB care capacity at the lowest-level facilities where patients most commonly started seeking care, and substantial heterogeneity in the duration and complexity of TB-related care pathways.

The most complex patient experiences and the most prolonged delays prior to treatment were associated with what we have termed interrupted evaluations. These pathways are those that include unusually long delays between the first relevant diagnostic procedure and TB diagnosis, as well as evidence that alternative causes of disease are being considered. Interestingly, our regression analysis showed that patients with DM were significantly less likely to experience interrupted evaluation, perhaps indicating an awareness among clinicians of DM as a risk factor for TB. However, patients whose care-seeking overlapped with the 2003 SARS epidemic, patients who had chronic lung disease or patients aged 65 years or above were all significantly more likely to experience interrupted evaluation. The SARS epidemic may have shifted attention towards alternative aetiologies of respiratory symptoms, as well as potentially generating increases in caseload that distorted usual practice. Comorbid chronic lung disease may have obscured TB symptoms, which would be unfortunate as chronic lung disease (eg, chronic obstructive pulmonary disorder) shares risk factors with TB and may causally increase the likelihood of TB and worsen outcomes.^22–24^ Lastly, differential diagnosis of TB may be more complex for older adults, and successful treatment is more challenging.^25^

In addition to service coverage, we were also able to analyse the referral flows of patients between different levels of the healthcare system. We found a general trend for escalation in level from initial care-seeking, with most patients seeking care at lower levels and most being treated at higher levels. While coverage of TB services was very high except at the lowest level, 36% of first attempts to seek care were still at facilities without the capacity to diagnose or treat TB. Patients did rapidly reach facilities where TB treatment was available, but 34% initiated treatment at a facility other than the first one they visited offering TB treatment, and the median delay between arriving at the facility where they were treated and treatment initiation was 6 days. These features may represent a lack of confidence or familiarity in diagnosing TB among clinicians at the primary care level. Further investigations to understand the reasons for this are warranted.

Our work builds on the PPA introduced in a series of recent papers.^7–13^ We were able to obtain similar outputs, including similar conclusions around coverage at the facility level typical of initial visits, but there are a number of differences which stem from our use of longitudinal individual-level data. Our coverage statistics for the availability of diagnosis at facilities are based not on direct facility data but on inferred availability from events in patient records. This will underestimate the actual coverage at facilities where tools are available but have not been used for patients in our data. This is likely to be particularly true at the lowest level of care. Importantly, our definition of ‘initial care-seeking’ differs from that used up to now in PPA, where initial care-seeking refers to patients’ preferred location to first access care for symptoms related to TB, determined from retrospective surveys.^7^ Our initial care-seeking events need not involve the initiation of care; indeed, multiple visits are typical between initial care-seeking and treatment initiation, including missed opportunities for earlier diagnosis. Our approach to identifying care-seeking related to a particular TB episode also differs from that used in Chen *et al*^19^ due to the potential for interrupted evaluation. This is why our median delay from initial visit to treatment (41 days) is somewhat longer than theirs (29 days) and longer than the median health system delay reported by a systematic review.^26^

Opportunities to validate our estimates are limited by their novelty. However, Taiwan CDC reported national treatment outcomes for 2010 as 71.1% treatment success and 20.2% death.^27^ Our analysis, based on a sample of cases in 2003–2010 classified 66.1% of pathways as ending with treatment completion and 14.2% with death. For both analyses, over 80% of deaths were among those aged 65 years.

The key strengths of this work lie in the use of individual-level data from a large population-representative sample, and the analyses of heterogeneity, referral patterns and determinants that this allowed. Due to the compulsory NHI system, we had data available to us relating to essentially all clinical encounters over a long period of time in individuals diagnosed with TB. Through our algorithm, we were able to identify early TB care-seeking events prior to diagnosis and analyse their patterns. It has been argued that the correct start-point for cohort-based analysis of patient care is the initial attempt to seek care rather than successful diagnosis or treatment initiation.^6^ However, this has previously relied predominantly on experiences reported retrospectively by patients,^26^ which are subject to recall biases. We were also able to link patient-level covariates, including comorbidities for risk factor analysis.

The main limitations of our analysis are around the nature of routine data, and the assumptions needed by any algorithm to interpret these records in terms of TB-related care-seeking. For example, our choice of time-out period to determine whether respiratory-related care-seeking events were part of a TB patient pathway affects how protracted and fragmented pathways are prior to diagnosis. The initial value of 60 days was based on a previous study evaluating health system delay of TB diagnosis in Taiwan.^19^ However, sensitivity analyses varying the choice of time-out showed that our main conclusions were not substantially affected. We relied on clinician coded ICD-9-CM codes to characterise clinical encounters; there may have been miscoding or omission of relevant secondary diseases for multimorbid patients. The choices of timings characterising treatments were based on standard treatment and may not have applied to extrapulmonary TB, although rates of extrapulmonary TB are low in Taiwan. In addition, our methods may work less well for TB patients with underlying chronic lung disease, for which they also interact with health services. Finally, our analysis cannot account for the delay between the start of infectiousness or symptoms and initial care-seeking. Prevalence-to-notification ratios in high TB burden settings typically correspond to a mean disease duration over a year,^28 29^ whereas the predominant approaches to measuring delays by asking patients typically result in shorter delays of the order 6 months.^26^ Our analysis sheds light on the health system delay contribution in this setting (from care-seeking to diagnosis) and suggests mean durations are strongly influenced by the few individuals with very long delays.

We developed our approach to analysing patient-level routine data using health insurance data for Taiwan, but this analysis framework could be adapted and applied to other settings and conditions. The prerequisite for this analysis is individual-level high-coverage routine data on healthcare use, ideally beyond services specific to the condition of interest. While such data are still relatively rare in many low-income and middle-income settings, health informatics systems are improving, for example, the wider use of electronic case-based notification systems for TB, and there are subnational linkage efforts that may allow such an analysis at a more local level.^30^ Use of different diagnostic codes for TB, different locally relevant comorbidities (eg, HIV) and clinical practices could all be included by adapting the open source code for our analysis. Likewise, consideration should be given to different time-out values as these are inevitably related to a system and patient characteristics. Future work building on our analysis for TB in Taiwan will include analysis considering health system costs and patient prescription charges, analysis with respect to proxy measures of socioeconomic status and developing simulation models replicating observed patterns of patient care-seeking.

## Conclusion

Our study demonstrates that longitudinal analysis of routine individual-level healthcare data can be used to generate a detailed picture of TB care-seeking pathways. This allows an understanding of several temporal aspects of care pathways, including lead times to care and the variability in patient pathways. Analysis of Taiwanese patient pathways suggests improved capacity to refer diagnostic specimens from peripheral healthcare facilities could simplify patient access to TB care.
