# Supplementary material for: Patient pathways of tuberculosis care-seeking and treatment: an individual-level analysis of National Health Insurance data in Taiwan
Source: BMJ Glob Health. 2020 Jun 21;5(6):e002187. doi: 10.1136/bmjgh-2019-002187 (PMC7307534; doi:10.1136/bmjgh-2019-002187)
Supplement: Supplementary data [file bmjgh-2019-002187supp001.pdf]

Definitions

A TB diagnosis and medications

TB case: An individual having 1) TB-related diagnosis codes (ICD-9-CM: 010-018) and 2) more than two anti-TB drugs prescribed for longer than 28 days or two consecutive 14 days prescriptions.

Regular treatment: An individual having 1) TB-related diagnosis codes (ICD-9-CM: 010-018) and 2) more than two anti-TB drugs prescribed for longer than 28 days (or two consecutive 14 days prescriptions).

First-line TB treatment: An individual having regular with a mixture of more than two first-line drugs: Isoniazid, Rifampicin, Pyrazinamide, Ethambutol, and Streptomycin.

Second-line TB treatment: An individual having regular with a more than two types of anti-TB drugs apart from above first-line drugs. To be noted that, a mixture of two first-line drugs and one second-line drug is a first-line TB treatment be the definition.

Treatment completion: An individual having taken regular treatments for more than 180 days.

Treatment initialisation, or empirical treatment: An individual having prescriptions of more than any two anti-TB drugs for shorter than 14 days.

Evaluation probably for TB: An individual having diagnostic or screening procedures that are specific to TB. The procedures are:

- Sputum culture test (Include Crystal, Parasite)
- Acid-fast culture
- Tuberculosis culture test
- Tuberculin Skin Test
- Chest X ray
- Chest computered tomography

Ku C-C, *et al. BMJ Global Health* 2020; 5:e002187. doi: 10.1136/bmjgh-2019-002187

Evaluation possibly for TB: An individual having medical procedures that can are highly relevant to TB suspicions. The procedures are:

- Bronchoscopy
- Mycoplasma pneumonia antigen test
- Streptococcus pneumonia antigen-latex agglutination
- Chlamydia pneumoniae antigen
- Pneumococcus antigen (urine)
- Legionella pneumophila antigen (urine)
- Cryptococcus antigen
- Aspergillus antigen
- Influenza A cystic fibrosis Antibody
- Influenza B cystic fibrosis Antibody
- Pleural fluid analysis

B TB-related illness:

Chronic lung disease, CLD: An individual having 1) respiratory diagnosis codes (ICD-9-CM: 460-510) and 2) drugs for respiratory syndrome prescribed for longer than 28 days.

Acute respiratory disease, ARD: An individual having 1) respiratory diagnosis codes (ICD-9-CM: 460-510) and 2) all drugs for respiratory syndrome prescribed shorter than 28 days.

Nontuberculous mycobacteria, NTM: An individual having NTM diagnosis codes (ICD-9-CM: 031)

C Comorbidities

Chronic lung disease, CLD: An individual having 1) respiratory diagnosis codes (ICD-9-CM: 460-510) and 2) drugs for respiratory syndrome prescribed for longer than 28 days.

Diabetes Mellitus, DM: An individual having 1) DM diagnosis codes (ICD-9-CM: 250, A181) and 2) DM control drugs prescribed for longer than 28 days. We cannot differentiate type 1 and type 2 diabetes in the National Health Insurance Research Database, NHIRD.

Human immunodeficiency virus, HIV: An individual having HIV related diagnosis codes (ICD-9-CM: 042, V08). We cannot differentiate HIV infection and acquired immune deficiency syndrome from the NHIRD.

Ku C-C, *et al. BMJ Global Health* 2020; 5:e002187. doi: 10.1136/bmjgh-2019-002187
