# Supplementary material for: Patient pathways of tuberculosis care-seeking and treatment: an individual-level analysis of National Health Insurance data in Taiwan
Source: BMJ Glob Health. 2020 Jun 21;5(6):e002187. doi: 10.1136/bmjgh-2019-002187 (PMC7307534; doi:10.1136/bmjgh-2019-002187)
Supplement: Supplementary data [file bmjgh-2019-002187supp002.pdf]

Individual Patient Pathway Analysis: Technical Guide

Chu-Chang Ku, Chien-Chou Chen, Simon Dixon, Hsien-Ho Lin, Peter J. Dodd

Overview

The objective of this appendix is to explain how to conduct an Individual Patient Pathway Analysis (IPPA) from individual healthcare data. This guidance is targeted for analysts to reason about and to implement the IPPA in their intended setting. The IPPA was developed using the information on TB patients in the National Health Insurance Research Database, Taiwan, though this guidance will indicate options to adapt to different data.

IPPA was designed to profile the efficacy of a healthcare system in responding to TB patients as well as to identify the obstacles of TB patients obtaining successful TB diagnosis and treatment. The IPPA approach was intended for use with passively and routinely collected healthcare utilisation data to inform the case seeking before patients being labelled as TB patients. Unfortunately, TB has many features overlapping with other respiratory diseases, such as pneumonia, and chronic lung disease. The presence of many non-TB related data entries is to be expected. In addition, healthcare utilisation data includes the use of diagnostic procedures and treatment, but the respective outcomes are not always available. These issues are dealt with during the IPPA implementation by inferring the results from subsequent events.

In general, the IPPA has two stages: patient pathways extraction and subsequent analysis of pathways. From the beginning, the data are usually collected with the perspective of healthcare providers in mind. The first stage of the IPPA translates the healthcare data to patient pathways, shifting information from provider-centred to patient-centred viewpoints. The extracted patient pathways are the first product of the IPPA (See S3 Algorithm 1). The second stage summarises and visualises the collected patient pathways.

The guidance is formulated as follows: Section 1 lists the terminology used during the IPPA. Section 2 prepares the IPPA ready data; Section 3 reads the data to a set of state-space dimensions; Section 4 trims the unnecessary information out and identifies care seeking episodes; Section 5 augments patient pathways by labelling stages of care seeking; Section 6 computes statistics from the patient pathways and Section 7 visualises the patient pathways step by step.

Ku C-C, *et al. BMJ Global Health* 2020; 5:e002187. doi: 10.1136/bmjgh-2019-002187

1. Terminology

<table>
<tr><th>Term</th><th>Definition</th><th>Example</th></tr>
<tr><td>Record</td><td>A healthcare record with variables of diagnosis, prescriptions, a visit time.

The diagnosis in the IPPA is the suggestion but not the actual health status.</td><td><table><tr><td>Time</td><td>Diagnosis</td><td>Medication</td></tr><tr><td>5</td><td>ARD*</td><td>None</td></tr><tr><td>10</td><td>ARD</td><td>Fast-acid test</td></tr><tr><td>20</td><td>TB</td><td>anti-TB drugs</td></tr></table>ARD: acute respiratory disease</td></tr>
<tr><td>Dimension</td><td>A state-space time-series linking a group of relevant records to inform the transition of state.</td><td>Related illness, capturing illness which can be initial consideration of a TB patient, or comorbidities with overlapped features of TB.

Evaluation, capturing the prescriptions of screening or diagnostic tools to identify TB.

Treatment, capturing the prescriptions of TB treatment</td></tr>
<tr><td>Value in dimension</td><td>A value reflects the information in the record given a dimension.

Zero value indicates there is no relevant record during a period of time.</td><td>In the Evaluation dimension,
Zero: no ongoing evaluation
Possible: diagnostic tools which might suggest TB
Probable: diagnostic tools which can identify TB</td></tr>
<tr><td>Time-out</td><td>The persistence of a healthcare record. The period will be extended if the next records contain similar information.</td><td></td></tr>
</table>

<table>
<tr>
<td>Episode</td>
<td>A collection of values of dimensions separated by periods with all dimensions equal to zero.

Non-TB episode: episodes with any of dimensions is not zero while none of the records during the episodes met the definition of confirmed TB.</td>
<td>
<table>
<tr><td>Time</td><td>Related illness</td><td>Evaluation</td><td>Treatment</td></tr>
<tr><td>105</td><td>ARD*</td><td>Zero</td><td>Zero</td></tr>
<tr><td>110</td><td>ARD</td><td>Possible</td><td>Zero</td></tr>
<tr><td>120</td><td>ARD</td><td>Possible</td><td>First line</td></tr>
<tr><td>...</td><td>...</td><td>...</td><td>...</td></tr>
</table>
*ARD: acute respiratory disease
</td>
</tr>
<tr>
<td>Patient pathway</td>
<td>An episode with a series of stages and the timing of progression.

Stage, indicating the progress of a pathway of a patient

Waiting, the patient not received and TB related medication yet after initial care seeking.
Evaluating, the patient is under evaluation but possibly for TB
Detecting, the patient is being considered as a potential TB patient.
Treating, the patient has been identified as a TB patient and initialised TB treatment.

State, specifying details of a stage. For example, the TB drug regimen</td>
<td>
<table>
<tr><td>Time</td><td>Stage</td><td>State</td></tr>
<tr><td>0</td><td>Waiting</td><td>Waiting</td></tr>
<tr><td>10</td><td>Evaluating</td><td>Evaluating</td></tr>
<tr><td>70</td><td>Evaluating</td><td>Interrupted Evaluation</td></tr>
<tr><td>90</td><td>Evaluating</td><td>Re-Evaluating</td></tr>
<tr><td>100</td><td>Detecting</td><td>Re-Detecting</td></tr>
<tr><td>120</td><td>Treating</td><td>First line treatment</td></tr>
<tr><td>300</td><td>End</td><td>Treatment completion</td></tr>
</table>
</td>
</tr>
</table>

Ku C-C, *et al. BMJ Global Health* 2020; 5:e002187. doi: 10.1136/bmjgh-2019-002187

2. Preparing IPPA ready data

The section guides how to manage data to which are ready to input to the IPPA. Three datasets (see figure), are required for the IPPA: patient information, healthcare facility information, and healthcare records. The patient information contains the basic variables of patients which are relevant to the decision making of either patients or healthcare providers. The healthcare facility information contains the type or the level of the facilities presented in the care-seeking records. The healthcare records, the most important dataset, contains the suggested clinical status (or diagnosis), prescriptions of diagnostic tools and medications, and keys referred to the patient and healthcare facility information.

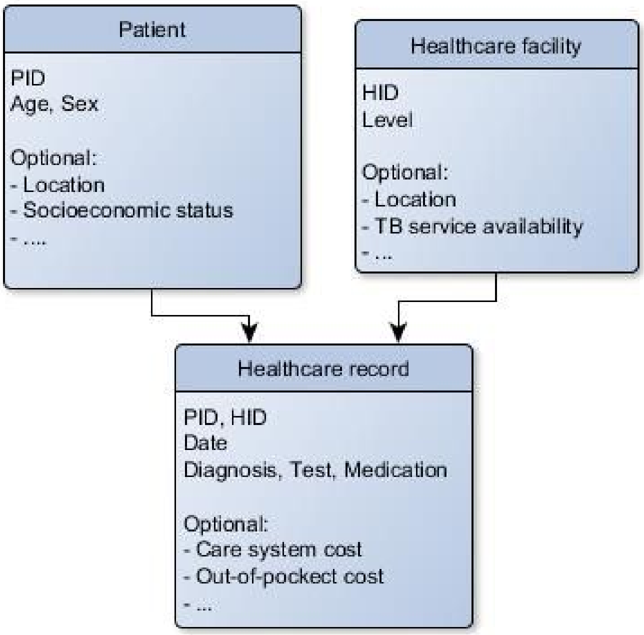

Patient information

Every patient entry requires a key for their identity (PID), linking to the healthcare records of the patient. Considering further analysis with the related variables, basic information, such as age and sex, are suggested. Other variables, such as socioeconomic status and comorbidities, can be included if of interest and available.

Healthcare facility (or hospital) information

Every hospital entry requires a key for its identity (HID), linking to the healthcare records issued in the hospital. Level of hospitals is a necessary variable in this dataset. The capacity of TB services can be included if available as an external hospital dataset. Otherwise, the IPPA considers if a service has been used in as hospital to indicate capacity of the service. Area-specific information can be considered if spatial diversity/disparity is of interest.

Healthcare records

Healthcare records are the central elements of the IPPA. Apart from the foreign keys linking to the patient and hospital data, the records should contain timing, diagnosis, evaluations, and treatments. The diagnosis includes TB diagnosis and that of related illness, comorbidities and diseases which could potentially be TB. The evaluations of TB should be ordered data based on their capabilities to identify TB. Note that the sensitivity and specificity of diagnostic tools might be different from setting to setting, as may the order of use. The previous appendix of definitions, Appendix A, lists the related diseases and medications considered in our application to the Taiwanese setting.

3. Transforming healthcare records into a three dimension system

(as S1 Algorithm, Algorithm 2)

A patient pathway considers three dimension of information: related illness, evaluation, and treatment. First, Related illness captures the illness which can be initial consideration of a TB patient, or comorbidities with overlapped features of TB. Second, Evaluation captures the prescriptions of screening or diagnostic tools to identify TB. Last, Treatment captures the prescriptions of TB treatment. Every record contains information in different domains, and some records can inform multiple domains. Therefore, this step is designed to pass the information in each record to the intended dimensions.

The IPPA builds three state-space dimensions based on the three domains. However, the data of healthcare utilisation might not contain the duration of state supported by each record. For example, TB culture can take several weeks to complete. The time to having the test result and following actions can be affected by the workload of the clinical laboratory as well as the schedule of the patient. As for anti-TB drug prescriptions, we can technically infer the duration through the number of drugs. However, how regularly the drugs are being taken depends on the implementation of DOTS and the schedule of patients to revisit hospitals. To correctly address the durations requires very detailed data. In such cases, the IPPA introduces a “Time-out” limit to presume the durations. In each domain, a specific value is set. As a default, we put 60 days for Related illness and Evaluation after visits and 30 days for Treatment after the drugs apparently ran out.

Starting from Zero state of each dimension, IPPA scans records iteratively. For every record, each dimension checks whether the record is relevant to it or not. If it is relevant, the dimension will transition the respective state. Before that, if the record occurs later than the Time-out period since the last relevant record, the dimension will transition to Zero. After scanning all records, the IPPA will check the end of the dimensions, considering the end of the data timespan and death time if present. See Algorithm 1 for the operational procedure.

Ku C-C, *et al. BMJ Global Health* 2020; 5:e002187. doi: 10.1136/bmjgh-2019-002187

4. Splitting Episodes and trimming unnecessary records off

(as S1 Algorithms, Algorithm 3)

In the data used - all the healthcare records over given a period - many non-TB care-seeking records are to be expected. However, they are necessary for completing the IPPA because some of them may be early events in the diagnosis of TB. As the previous step has joined the records in the same domain to formulate the system of three dimensions, this step is going to join the non-zero states in different domains.

Once the system of three dimensions aligned, we can locate the periods with all dimensions equal to zero. The step splits the three dimensions by these periods to specify episodes. For the separated episodes, we identify the episodes have confirmed TB (in our demonstration, ICD-9-CM codes for TB and more than two types of anti-TB drugs prescribed over 28 days), and drop the others. Each of the kept episodes will be formulated as patient pathways thereafter.

This step allows construction of episodes that included records related to care-seeking prior to TB diagnosis as possible. Also, it trims the healthcare records which are not relevant to TB care off. To be noted that, whether an episode is TB related or not is affected by the length of the “Time-out” period. For a short “Time-out”, an episode will be fragmented as several episodes. The care-seeking before TB diagnosis will be ill-addressed. For a long “Time-out”, TB episodes will be mixed with irrelevant episodes. (See the appendix of sensitivity analysis).

Ku C-C, *et al. BMJ Global Health* 2020; 5:e002187. doi: 10.1136/bmjgh-2019-002187

5. Finishing patient pathway construction

(as S1 Algorithms, Algorithm 4)

A TB episode includes the dynamics of the three state space dimensions but not the meaning of them. This step concludes the three dimensions into a patient pathway. For every separated TB episodes, this step. The stages and states in patient pathways should summarise by not only the current states of the three dimensions but also the states before and after. For example, the "Re-Evaluating" state indicates (1) evaluations are going on, (2) previous evaluations were interrupted, and (3) TB treatment has not started yet. As in the following definition, this step labels care-seeking stages considering the states of the three dimensions.

Waiting Stage

In Waiting Stage, the patients have started their care-seeking at hospitals but are not considered as potential TB patients. Namely, they are "waiting" for TB-related evaluation.

State	Dimensions*	Contexture	Note
Waiting	R: non-zero<br>E: Zero<br>T: Zero	Start with initial care-seeking	Waiting for the first TB-related evaluation or treatment.

* R: related illness, E: evaluation, T: treatment

Evaluating Stage

In the Evaluating Stage, physicians start to use evaluation techniques for the patients. TB might not be a consideration in the evaluation, but the techniques should be able to narrow the possibilities down until TB-specific evaluations prescribed.

State	Dimensions*	Contexture	Note
Evaluating	E: Possibly<br>T: Zero	No IE before	Under evaluations which can narrow the possibility down to TB
Interrupted Evaluation (IE)	R: non-zero<br>E: Zero<br>T: Zero	After a period with the evaluation dimension in a non-zero state	The previous evaluation does not narrow the possibility down to TB which might be because of (1) comorbidity, (2) false negative, or (3) self-referral.
Re-Evaluating	E: Possibly<br>T: Zero	Have IE before	

* R: related illness, E: evaluation, T: treatment

Ku C-C, et al. BMJ Global Health 2020; 5:e002187. doi: 10.1136/bmjgh-2019-002187

TB Detecting Stage

In TB Detecting Stage, the evaluation techniques which can identify TB if clinicians are well-trained. Apart from the techniques, anti-TB drugs with doses below regular regimens can be considered in this stage as well, namely, empirical treatment or treatment initialisation.

State	Dimensions*	Contexture	Note
TB-Detecting	E: Probably<br>T: not meet 1st line or 2nd line	No IE before	Being evaluated by procedures which can identify TB (Evaluations probably for TB)
Interrupted Evaluation (IE)	R: non-zero<br>E: Zero<br>T: Zero	After period with the evaluation dimension in a non-zero state	IE can be a state in Evaluating Stage or TB-Detecting Stage. It depends on the most TB specific evaluation used before.
Re-Detecting	E: Probably<br>T: Zero	Have IE before	Re-visiting TB-Detecting State after Interrupted Evaluation

* R: related illness, E: evaluation, T: treatment

Treating Stage

Once TB confirmed, Treating Stage will start. The stage captures the intensity of treatment used.

State	Dimensions*	Contexture	Note
First-line treatment	T: 1st line	Between two periods with the treatment dimension in non-zero states	Being treated with first-line TB regimen.
Treatment change	R or E: non zero<br>T: see contexture	A zero treatment dimension between two periods of treatment dimension in non-zero states; or the timing when treatment intensity increases.	Switching between two TB treatments or temporal treatment interruption. Could be zero duration.
Second-line treatment / retreatment	see contexture	T: 1st line after Treatment change or T: 2nd line	Being treated with second-line TB regimen or any regimen after Treatment change.

Treatment Outcome

Treatment Outcomes are the end of patient pathways. The labels are totally customizable for different settings Datasets of treatment outcome and death registration outside healthcare records can be linked if available.

In our demonstration setting, registering in the National Health Insurance programme in Taiwan is compulsory. We used leaving the programme as the indicator of death.

Outcome	Definition
Treatment completed	> 180 days of treatment period
Censored	Reach the end of data
Dead	Leaving the National Health Insurance programme.
Lost to follow-up (LTFU)	Other

Ku C-C, *et al. BMJ Global Health* 2020; 5:e002187. doi: 10.1136/bmjgh-2019-002187

6. Statistics and indices of the Individual Patient Pathway Analysis

Hospital-level of initial care-seeking features the places to start care-seeking for the patients with TB symptoms. The levels of hospitals or facilities are defined differently setting by setting. Our demonstration only presents four levels while it can be disaggregated by private and public sectors, by hospital divisions, or by counties. In Taiwan, the National Health Insurance covers both private and public sectors with the same services, so we did not separate them.

Coverage of TB services denotes the percentage of hospitals have TB services available. The TB services are the evaluation tools with respect to the medications in Evaluating, TB Detecting, and Treating stage of patient pathways. The denominator is the number of hospitals has ever been visited in the collected patient pathways; the numerator is the number of hospitals has ever provided respective TB services during the data timespan. In the original IPPA, the index is measured by whether the hospitals can provide respective TB services. That might overestimate the values if the services are not considered in practice. On the contrary, our approach might underestimate the values in the case of the hospitals can provide the services but have not met the situations to use. We suggest reporting values from both approaches if possible as lower and upper bounds.

Hospital-level of stages denotes the distributions of hospital levels at the starts of stages. The options are the same as **Hospital-level of initial care-seeking.**

Hospital-level of notification features the places which notify TB cases to authorities. In the setting of Taiwan, and so our demonstration, the notification process and treatment prescribing are synchronised. The distribution was the same as **Hospital-level of Treatment Stage**, so we did not show them separately.

Accessibility to TB services at initial care seeking denotes the percentage of patient pathways which have initial care seekings at a hospital which has TB services available (evaluations or treatments). With the individual hospital data identifiable, it is calculated by (1) linking initial care-seeking records to the availability of the corresponding hospitals, and (2) finding records can access to TB services within all initial care-seeking records. However, if the data does not support individual hospitals, applying the original approach in the PPA [1] is suggested.

Time to arriving at a hospital with TB treatment denotes when is the first time seeking care at a hospital which can provide TB treatment. The period starts with the time of initial care-seeking. Again, the availability of TB treatment in our demonstration was based on whether the hospital has provided TB treatments or not during the timespan.

Time to arriving at the hospital initialise TB treatment denotes when is the first time seeking care at the hospital which ultimately provides TB treatment to the patient.

System Delay, time to treatment start, denotes the length of the period between the initial care-seeking and the start of regular TB treatment. This is the common output of care-seeking delay studies. However, the definitions may be different for different data sources.

No System Delay indicates a patient pathway starts with regular TB treatment prescription at the initial care-seeking. We suggested reporting this and System Delay and a proportion

Interrupted Evaluation indicates at least one Interrupted Evaluation in a patient pathway. The presence shows if the diagnosis has been put on hold, potentially in favour of other investigations. The percentage of Interrupted Evaluation quantifies the complexity of TB diagnosis in a healthcare system. Applying covariate analysis can identify the risk factors related to interruption. The presence of Interrupted Evaluation has multiple meanings. Firstly, comorbidities, especially chronic lung diseases, can mask the features of TB. As the respiratory symptoms of TB are not unique, the clinicians might not aware active TB from patients who have had those symptoms already. Secondly, there are competing diseases to be considered before diagnosing a patient as a TB patient. For example, in the Western Pacific region, TB is prevalent in the old population and is sometimes similar to pneumonia. Considering the higher fatality rate of pneumonia than TB, TB could be less urgent. Thirdly, the false negative of any TB test results and false positive of non-TB test results might happen. Either of them can cause interrupted evaluation.

System cost, out-pocket cost, and healthcare contacts (optional) in each stage measure the burden for the perspectives of a healthcare system and patients. Using the records grouped by the stage when they occurred, these three indices are summarised. The number of healthcare contacts is a proxy of the time cost of a patient pathway during a stage. This should be available since it corresponds to the input of the IPPA, but the other two depend on availability. Additional costing survey is suggested to provide more precise measures and to broaden the IPPA.

7. Visualisation

Accessibility and Coverage

The figure (as Fig 2A in the paper) is adapted from the original PPA. Seven columns are included in the diagram. The A-1 shows the places of initial care-seeking by hospital level. A-2.1 shows the coverages of evaluation services in proportions by hospital level. This is from the perspective of healthcare providers. A-2.2 shows the accessibility at initial care-seeking. This is from the perspective of patients, measuring if the patients seek care at proper places (See Statistics and Indices). A-3 and A-4 replicate A-2 but targeted at Evaluations probably for TB and treatment.

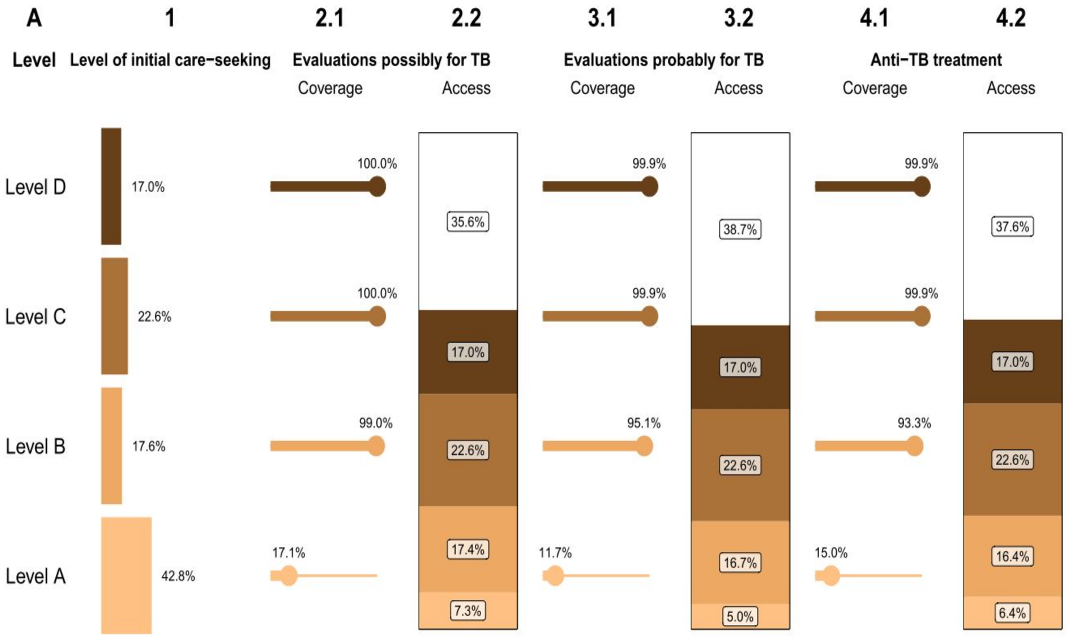

Referral Flow

Figure 2 (as Fig 2B in the paper) shows an example of the referral flow diagram. The diagram is a Sankey diagram describing the progress in hospital levels during care-seeking. Four columns match the four stages of patient pathways: Waiting, Evaluating, TB Detecting, and Treating. The blocks in each column are the hospital levels in each stage. For a pathway, the hospital level in a stage is based on the hospital of the first care-seeking in the stage. While some pathways do not have all four stages, the diagram uses the start of the next stage for the current stage. For example, a pathway started with TB-Detecting Stage at a Level B hospital. The hospital levels in Waiting and Evaluating Stages are Level B as well. The heights of the blocks are determined by the counts of the pathways. The ribbons between columns link the current hospital levels to the hospitals triggering the next stages.

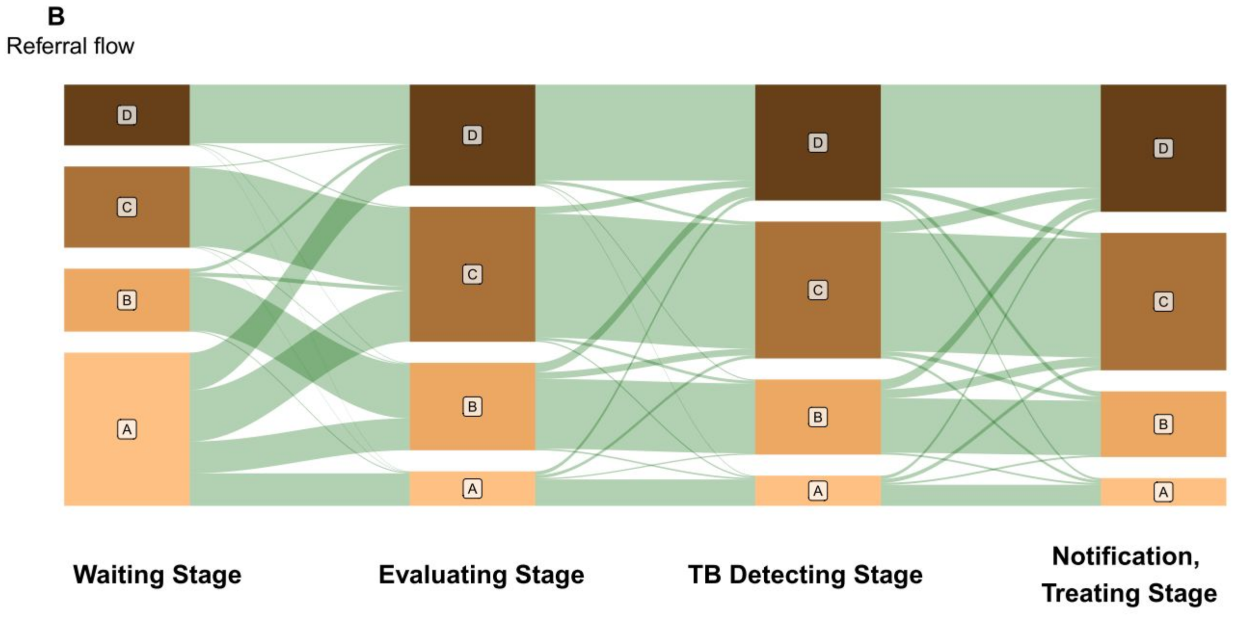

Care-seeking Gap

Figure 3 (as Fig 3 in the paper) features the gaps between treatment accessible and treatment start. Three checkpoints are considered. The first is when a patient arrived at a hospital with TB treatment. From this checkpoint, the patient starts having a chance to be under TB treatment. The second is when a patient arrived at the hospital which provides TB treatment for them ultimately. From this checkpoint, the inter-hospital referral has been completed in the pathway. The last is when TB treatment starts. The curves are the cumulative percentage of pathways that have reached the respective checkpoints. The difference between the first and the second checkpoints (and their curves) can be regarded as a care-seeking gap due to inter-hospital referrals. The difference between the second and the third checkpoints can be regarded as a gap due to intra-hospital referrals and diagnostic dimensions.

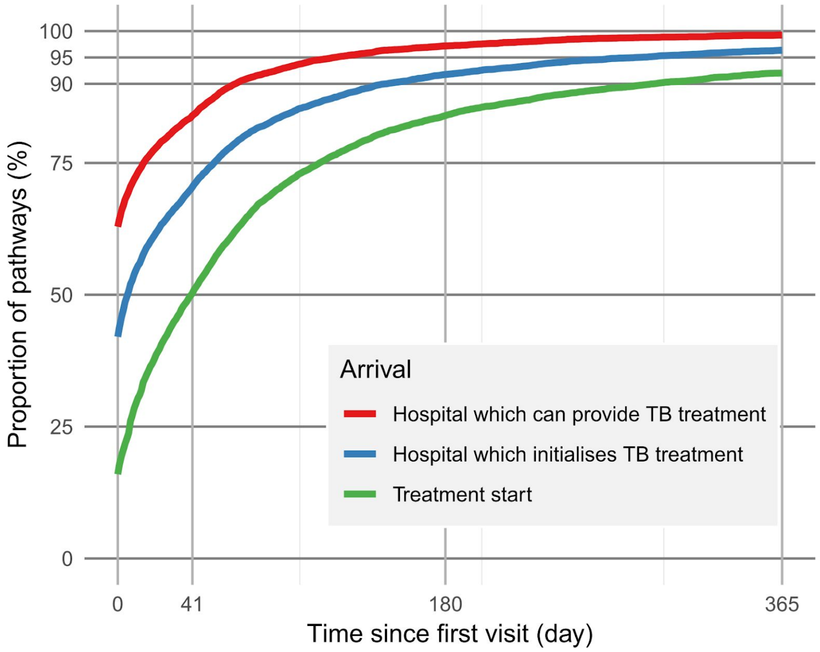

Pattern Frequency

Figure 4 visualise the patterns of patient pathways before and after treatment start. The pattern means the series of states in a pathway without considering their timings in the stages. For example, a pathway starting Waiting state at day 0, Evaluating state at day 30, Treating state at day 40 has the pre-treatment pattern of “Waiting-Evaluating-Treating”. In the figures, each horizontal bar represents a pattern; the height indicates the number of pathways having this pattern; the width indicates the proportion of time spent in each state on average. The tiny rectangles on the right indicate the last states of patterns while the widths are meaningless. The blink bar in the pre-treatment figure quantifies the pathways having treatment at initial care-seeking. The bars are sorted by the numbers of pathways in the respective patterns. The figures are used to highlight the heterogeneity of the pathways.

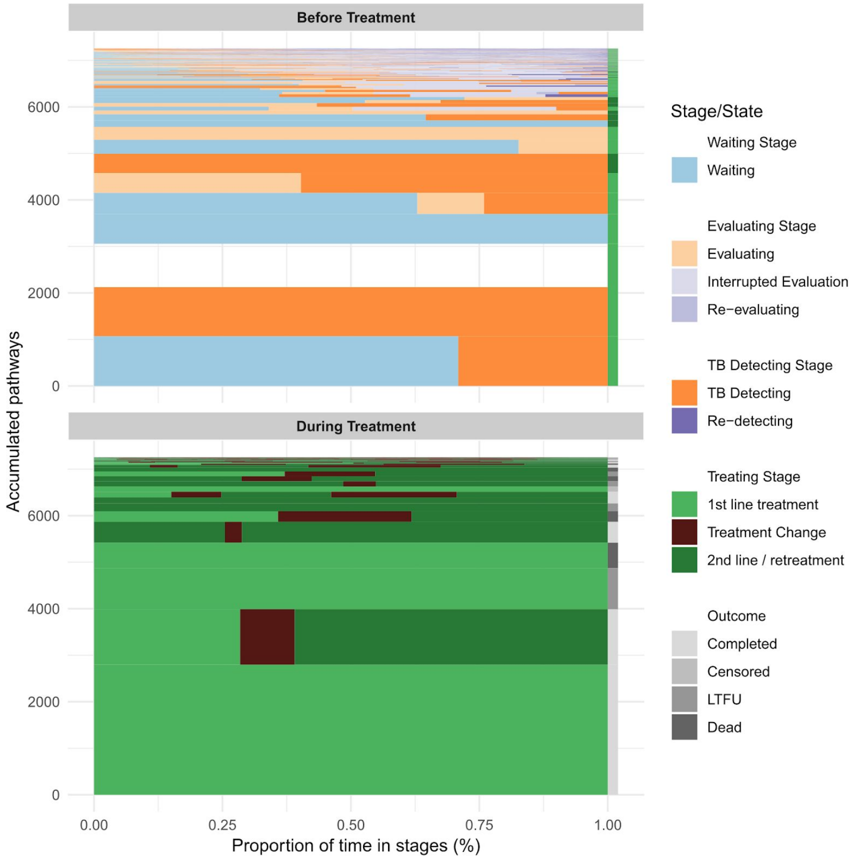

Stage Distribution

Figure 5 visualises the distribution of states of patient pathways. Aligning patient pathways by the time of initial care-seeking, every column in the figure indicates state distribution in one day; the height in each block indicates the number of pathways in the state at the day; for the pathways ended before the day, the treatment outcomes are extended to the day. The figure is used to understand the time spent in each state and to know the accumulation of treatment outcomes. For our example, the Interrupted Evaluation formed a flat tail, which dying out slowly, in the figure.

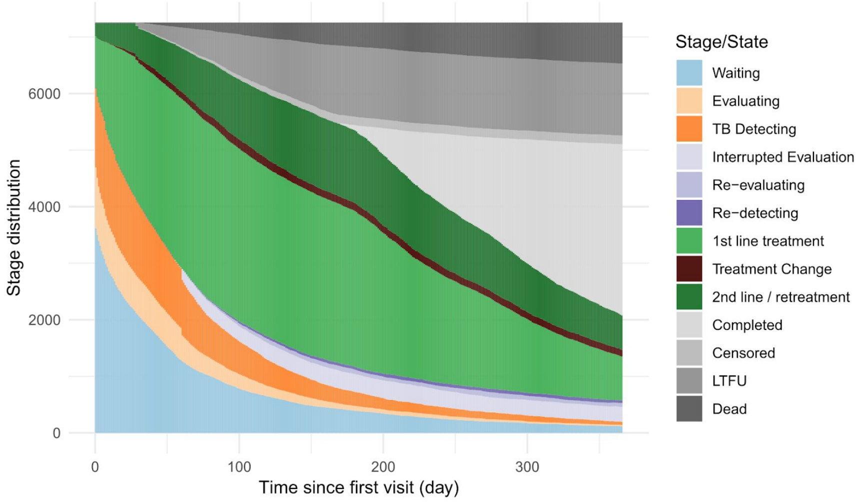

Reference

1. Hanson CL, Osberg M, Brown J, Durham G, Chin DP. Conducting Patient-Pathway Analysis to Inform Programming of Tuberculosis Services: Methods. J Infect Dis. 2017;216: S679–S685.

Ku C-C, *et al. BMJ Global Health* 2020; 5:e002187. doi: 10.1136/bmjgh-2019-002187
