# Supplementary material for: Patient pathways of tuberculosis care-seeking and treatment: an individual-level analysis of National Health Insurance data in Taiwan
Source: BMJ Glob Health. 2020 Jun 21;5(6):e002187. doi: 10.1136/bmjgh-2019-002187 (PMC7307534; doi:10.1136/bmjgh-2019-002187)
Supplement: Supplementary data [file bmjgh-2019-002187supp003.pdf]

Algorithms

Algorithm 1: Patient pathway extraction: overview

```
Data: records: an array of healthcare records of an individual
      TOR: Time-out of Related illness
      TOE: Time-out of Evaluation
      TOT: Time-out of Treatment
Result: Patient pathways of a TB patient
/* Initialise dimensions */;
rel ← New Related illness dimension;
eva ← New Evaluation dimension;
tre ← New Treatment dimension;
/* Read all records in each dimension */;
rel.readRecords(TOR, records);
eva.readRecords(TOE, records);
tre.readRecords(TOT, records);
/* Collect dimensions and cut them into episodes */;
episodes ← cutEpisodes(rel, eva, tre);
pathways ← ∅ /* Initialise a empty set collecting pathways*/;
foreach episode ∈ episodes do
    pathway ← formulatePathway(episode);
    pathways.append(pathway)
end
return pathways;
```

See *dim*.readRecords(...) in Algorithm 2
See cutEpisodes(...) in Algorithm 3
See formulatePathway(...) in Algorithm 4

Ku C-C, *et al. BMJ Global Health* 2020; 5:e002187. doi: 10.1136/bmjgh-2019-002187

Algorithm 2: Read records in each dimension (*dim*.readRecords(...))

Data: *dim*: dimension, *rel*, *eva*, or *tre*
timeout: timeout for the selected dimension
records: an array of healthcare records of an individual
Result: the transition history in the dimension

/* Initialise the dimension with a Null event */;
$time_{curr} \leftarrow 0$;
$time_{wait} \leftarrow \infty$ /* the end of waiting time */;
state $\leftarrow$ *Null*;
Initialise *dim* with *state* at $time_{curr}$;
foreach *record* $\in$ *records* **do**
    $time_{curr} \leftarrow$ time of *record*;
    if $time_{curr} > time_{wait}$ **then**
        /* Reset state */;
        $time_{wait} \leftarrow \infty$ /* the end of waiting time */;
        state $\leftarrow$ *Null*;
        dim transits to *Null* at $time_{wait}$;
    end
    if *record is relevant to dim* **then**
        $time_{wait} \leftarrow time_{curr} + timeout$;
        if *record can progress state* **then**
            /* Progress */;
            state $\leftarrow$ the matched state of *record*;
            dim transits to *state* at $time_{curr}$;
        end
    end
end
/* Close record reading */;
dim transits to *Null* at $time_{wait}$;

Ku C-C, *et al. BMJ Global Health* 2020; 5:e002187. doi: 10.1136/bmjgh-2019-002187

Algorithm 3: Collect dimensions and cut them by periods without events ($cutEpisodes(...)$)

```
Data: rel: state history in related illness dimension
      eva: state history in evaluation dimension
      tre: state history in treatment dimension
Result: A set of care seeking episodes
ts ← ∅ /* A collection storing state transition times */;
foreach dim ∈ [rel, eva, tre] do
    forall State transition time t of dim do ts.add(t);
end
Remove duplicated time points in ts Sort ts (ascending);
episodes = ∅ /* A collection for storing episodes */;
foreach t ∈ ts do
    if all[rel, eva, tre] are Null at t then
        /* Separate state-transition history*/;
        x ← state history before t split from rel;
        y ← state history before t split from eva;
        z ← state history before t split from tre;
        /* Join dimensions */;
        episode ← [x, y, z];
        episodes.append(episode);
    end
end
return episode;
```


Ku C-C, *et al. BMJ Global Health* 2020; 5:e002187. doi: 10.1136/bmjgh-2019-002187

Algorithm 4: Patient pathway formulation ($formulatePathway(...)$)

Data: *episode*: an episode with state transition history in rel, eva, and tre
Result: A patient pathway
/* Identify key information */;
$t_{eva} \leftarrow$ time of first evaluation possibly for TB;
$t_{det} \leftarrow$ time of first evaluation probably for TB;
$t_{tre} \leftarrow$ time of the start of first regular TB treatment;
/* Group state transition history */;
$history_{eva} \leftarrow$ history between t_{eva} and t_{det};
$history_{det} \leftarrow$ history between t_{det} and t_{tre};
$history_{tre} \leftarrow$ history after t_{tre};
/* Start to construct pathway */;
$pathway \leftarrow \emptyset$ /* Initialise patient pathway */;
$state \leftarrow$ initial state of *episode*;
put *state* into *pathway* ;
$ie \leftarrow False$ /* indicating had interrupted evaluation or not */;
/* Read state series in **Evaluating Stage** */;
foreach $dimensions \in history_{eva}$ **do**
  $state \leftarrow$ find an state in **Evaluating Stage** matched *dimensions* and *ie*;
  if *state is* ***Interrupted Evaluation*** **then** $ie \leftarrow True$;
  put *state* into *pathway* ;
end
/* Read state series in **TB Detecting Stage** */;
foreach $dimensions \in history_{det}$ **do**
  $state \leftarrow$ find an state in **TB Detecting Stage** matched *dimensions* and *ie*;
  if *state is* ***Interrupted Evaluation*** **then** $ie \leftarrow True$;
  put *state* into *pathway* ;
end
/* Read state series in **Treating Stage** */;
$state \leftarrow$ find the initial treatment level in $history_{tre}[0]$;
put *state* into *pathway* ;
foreach $dimensions \in history_{tre}[0:]$ **do**
  $state \leftarrow$ find the treatment level in *dimensions*;
  if *treatment level increased* **then**
    put **Treatment Change** into *pathway* ;
  end
  put *state* into *pathway* ;
end
/* Finalise patient patient formulation*/;
$state \leftarrow$ find the treatment outcome ;
put *state* with the time of treatment end into *pathway*;
return *pathway*;

Ku C-C, *et al. BMJ Global Health* 2020; 5:e002187. doi: 10.1136/bmjgh-2019-002187
