# Supplementary material for: Patient pathways of tuberculosis care-seeking and treatment: an individual-level analysis of National Health Insurance data in Taiwan
Source: BMJ Glob Health. 2020 Jun 21;5(6):e002187. doi: 10.1136/bmjgh-2019-002187 (PMC7307534; doi:10.1136/bmjgh-2019-002187)
Supplement: Supplementary data [file bmjgh-2019-002187supp004.pdf]

Description	programming language	URL
Online demonstration		https://patientpathwayanalysis.github.io/
Example data		https://github.com/PatientPathwayAnalysis/IPPA-data
Pathway extraction	python 3.6	https://github.com/PatientPathwayAnalysis/IPPA-py
Visualisation	R 3.5.1, ggplot2	https://github.com/PatientPathwayAnalysis/IPPA-vis
Visualisation-d3	javascript, D3.js v3	https://github.com/PatientPathwayAnalysis/IPPA-d3
