# Supplementary material for: Patient pathways of tuberculosis care-seeking and treatment: an individual-level analysis of National Health Insurance data in Taiwan
Source: BMJ Glob Health. 2020 Jun 21;5(6):e002187. doi: 10.1136/bmjgh-2019-002187 (PMC7307534; doi:10.1136/bmjgh-2019-002187)
Supplement: Supplementary data [file bmjgh-2019-002187supp005.pdf]

Sensitivity analysis

In the individual patient pathway analysis (IPPA), a “time-out” system is applied to define whether two healthcare records is related or not. Here, we tested the sensitivities of Time-out for Related illness domain (TOR) and Time-out for Evaluation domain (TOE). We varied them from 30 days to 120 days by 30 days and (TOR, TOE) = (60 days, 60 days) were used as a comparator.

Throughout the sensitivity analysis, we will use two types of diagrams to demonstrate how the selected indices reflect on the time-out changes. The first uses heatmaps, mapping the indices given different time-out values (i.e. Figure 1). The second is barplot (or unordered tornado chart) which shows the marginal changes of the indices while increasing or decreasing by 30 days (i.e. Figure 2). The changes were measured by

Difference $N_{tor,toe} - N_{60,60}$ or

Change rate $\frac{N_{tor,toe} - N_{60,60}}{N_{60,60}} \times 100\%$,

depending on variable types.

Ku C-C, *et al. BMJ Global Health* 2020; 5:e002187. doi: 10.1136/bmjgh-2019-002187

1 Number of pathways

This section assesses the number of pathways rendered by the IPPA with different TOR and TOE. The changes were measured by the change rate. Figure 1 shows that the number of pathways is negative corrected with both TOR and TOE. The changes were within 5%. Figure 2 shows that increasing TOR and TOE caused higher change rates than decreasing while the number of pathways is more sensitive to TOE.

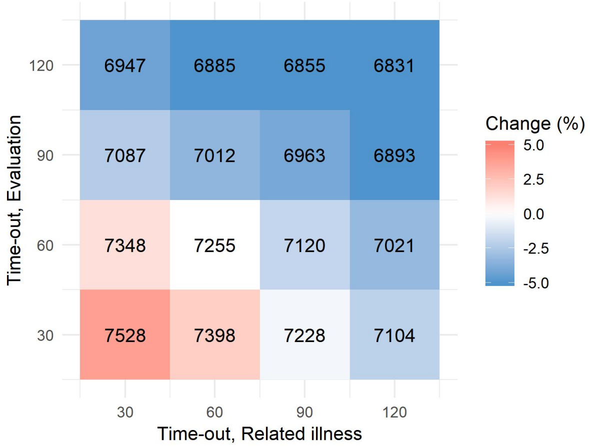

Figure 1: Heatmap: Number of Pathways

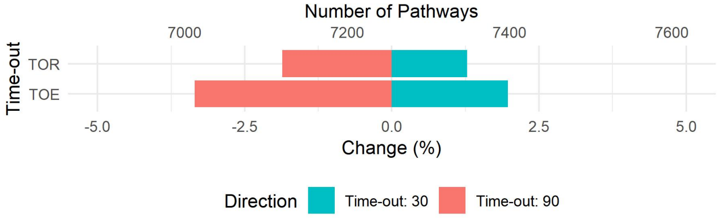

Figure 2: Marginal changes: Number of Pathways

Ku C-C, *et al. BMJ Global Health* 2020; 5:e002187. doi: 10.1136/bmjgh-2019-002187

2 Length

This section assesses the length of patient pathways.

- **Number of Contacts, median**: the length considering how many health-care contacts happened during the patient pathways started from initial care-seeking to treatment end.
- **System Delay, median**: the duration from initial care-seeking to treatment start.
- **Pathway Length, median**: the duration from initial care-seeking to treatment end.

These indices were summarised by median, and the changes were measured by change rate.

As Figure 3 shows, the numbers of contact ranged from 16 to 25 while the values were positively correlated to TOR and TOE. Figure 4 addresses the system delays, showing the values were sensitive to both TOR and TOE. When TOR and TOE were both 120 days, the change is more than being doubled compared with 60 days. Figure 5 focuses on the pathway lengths. Figure 6 summarises the sensitivity of the length of pathways. The system delay was the most sensitive to TOR and TOE, while the total pathway length was the last. TOR and TOE had equal impacts on the number of contacts in this analysis. Increasing TOR and TOE brought more changes than decreasing across these three indices. The high sensitivity of the median system delay suggested an external validation with future interview data in the same setting. A previous study, Chen et al. [1], estimated the system delay in Taiwan was 29 days (interquartile range 5–73) with the same database and TB definition as my study. Although their assessment did not consider interrupted evaluations and patients having chronic lung conditions, and so their estimates constitute a lower-bound for our approach. Comparing with other settings, Sreeramareddy et al. [2] summarised 52 studies, finding the system delays to TB treatments ranged from 2 to 87 days, finding that the low-income and high-income settings did not have a significant difference. However, the retrieved studies in their review showed an imbalance in that the studies with longitudinal data were conducted in specific hospitals or sub-populations, while the studies that covered the general population were cross-sectional. My study, which used longitudinal data on the general population, therefore, cannot be compared with them directly. Therefore, I suggest using a retrospective design with interviewing patients embedded in the longitudinal data. This approach can validate the lengths of patient pathways from the IPPA and highlight the difference from perspectives of patients and the health system.

Ku C-C, *et al. BMJ Global Health* 2020; 5:e002187. doi: 10.1136/bmjgh-2019-002187

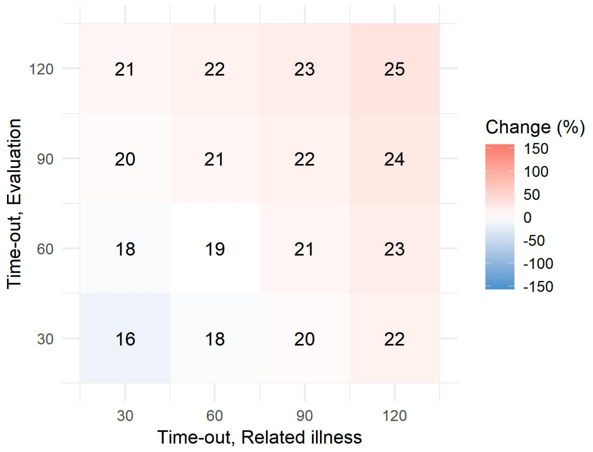

Figure 3: Heatmap: Number of contacts in median

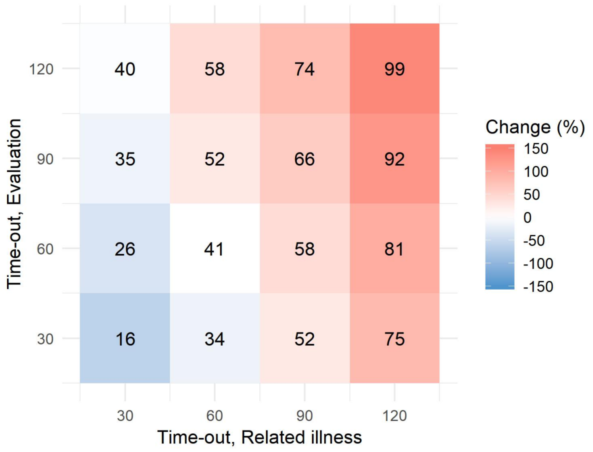

Figure 4: Heatmap: System delay in median

Ku C-C, *et al. BMJ Global Health* 2020; 5:e002187. doi: 10.1136/bmjgh-2019-002187

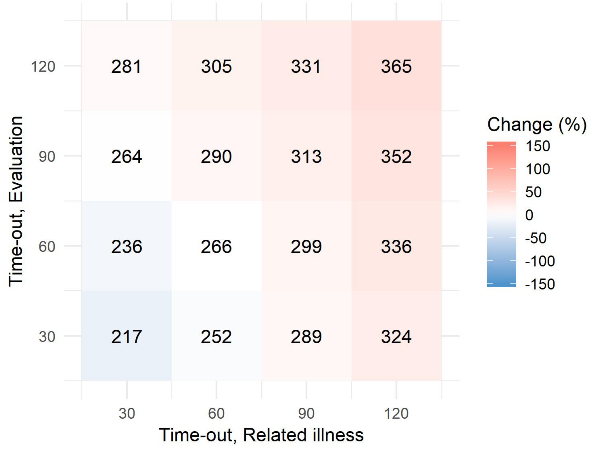

Figure 5: Heatmap: Length of Pathways in duration

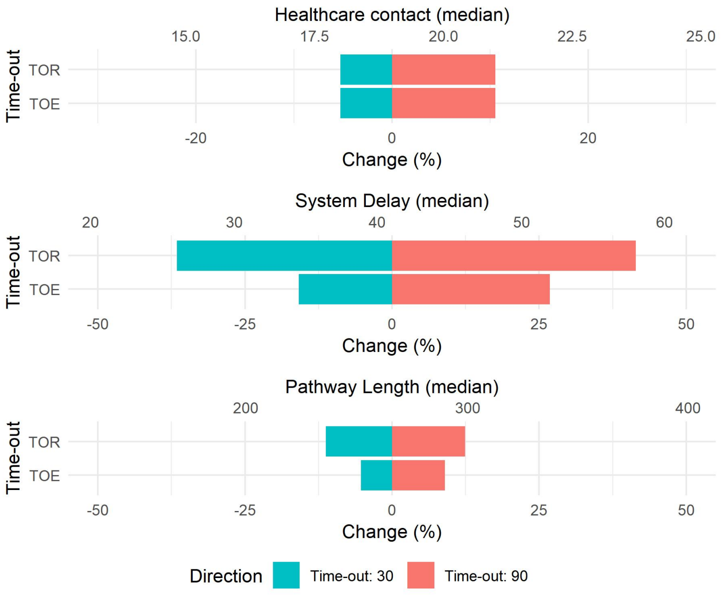

Figure 6: Marginal changes: Length of Pathways

Ku C-C, *et al. BMJ Global Health* 2020; 5:e002187. doi: 10.1136/bmjgh-2019-002187

3 Typology

This section assesses the typology of patient pathways.

- **Initialised at Level A Hospital**: whether the initial care-seeking of pathways were in Level A hospitals.
- **Interrupted Evaluation**: if the pathways experienced interrupted evaluation.
- **Zero Delay**: indicates if the pathways started their treatment at the day of initial care-seeking.

These indices were summarised by proportion, and the changes were measured by difference.

As Figure 7 shows, **Initialised at Level A Hospital** were no sensitive to TOE while that and TOR were positively correlated. Figure 8 highlights higher TOR led more **Interrupted Evaluation** but TOE had negative influence. **Zero Delay** in Figure 9 shows negative correlations with TOR and TOE while the two Time-outs were equally contributed. Figure 10 summarises the sensitivity of the typology of pathways. The marginal changes were usually smaller than 5%. However, **Interrupted Evaluation** was very sensitive to TOR and TOE compared with the other two indices.

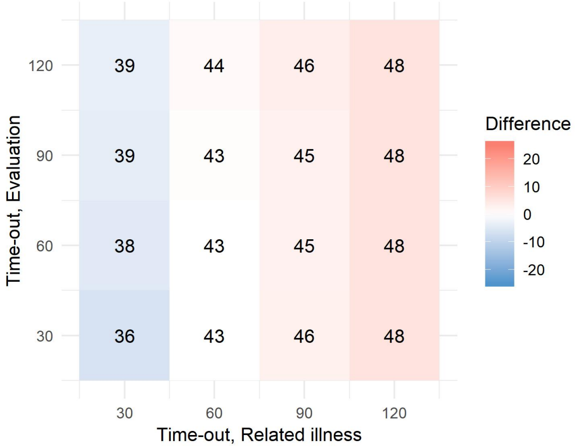

Figure 7: Heatmap: Initialised at Level A hospital (%)

Ku C-C, *et al. BMJ Global Health* 2020; 5:e002187. doi: 10.1136/bmjgh-2019-002187

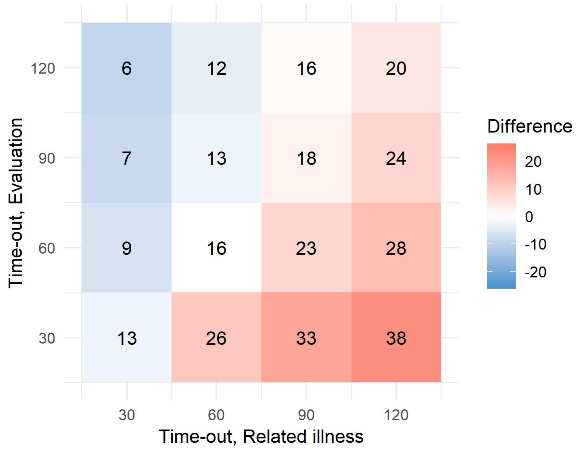

Figure 8: Heatmap: Interrupted Evaluation (%)

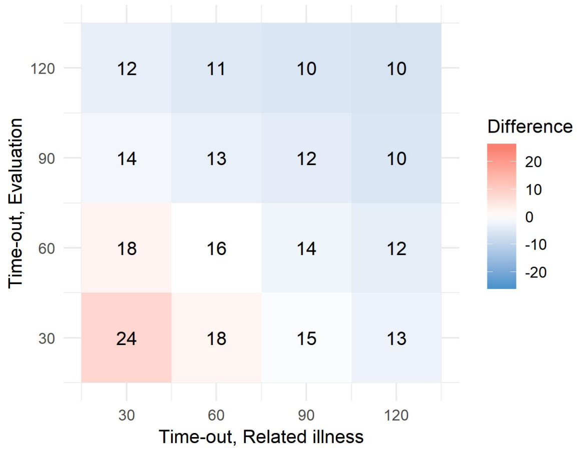

Figure 9: Heatmap: Zero Delay (%)

Ku C-C, *et al. BMJ Global Health* 2020; 5:e002187. doi: 10.1136/bmjgh-2019-002187

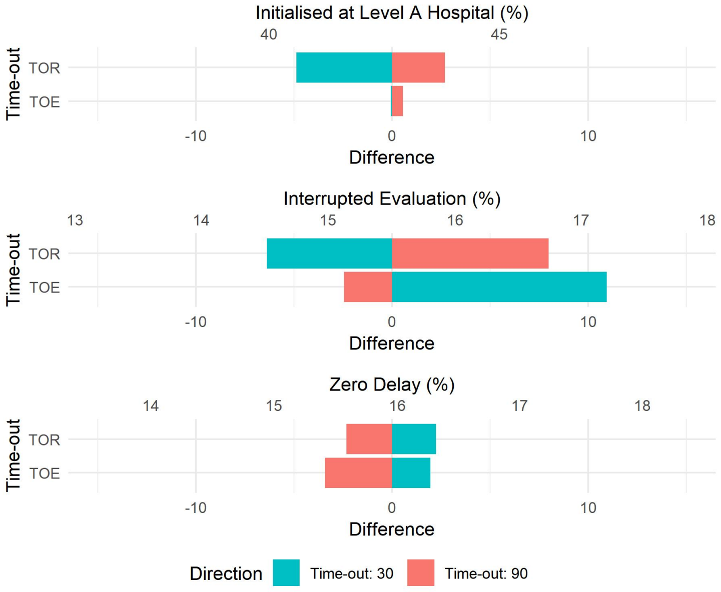

Figure 10: Marginal changes: Typology

References

[1] Chen CC, Chiang CY, Pan SC, Wang JY, Lin HH. Health system delay among patients with tuberculosis in Taiwan: 2003-2010. BMC Infect Dis. 2015 Nov;15(1):491.

[2] Sreeramareddy CT, Panduru KV, Menten J, Van den Ende J. Time delays in diagnosis of pulmonary tuberculosis: a systematic review of literature. BMC Infect Dis. 2009 Jun;9(1):91.
