# Supplementary material for: Patient pathways of tuberculosis care-seeking and treatment: an individual-level analysis of National Health Insurance data in Taiwan
Source: BMJ Glob Health. 2020 Jun 21;5(6):e002187. doi: 10.1136/bmjgh-2019-002187 (PMC7307534; doi:10.1136/bmjgh-2019-002187)
Supplement: Supplementary data [file bmjgh-2019-002187supp006.pdf]

Stage	State	Description
Waiting	Waiting	Waiting for first TB-related evaluation or treatment
Evaluating	Evaluating	Being evaluated by procedures which can narrow the possibility down to TB (Evaluations possibly for TB)
	Interrupted Evaluation (IE)	Previous evaluations do not narrow the possibility down to TB because of (1) comorbidity, (2) false negative, or (3) self-referral.
	Re-Evaluating	Visiting **Evaluating State** after **Interrupted Evaluation**
TB-Detecting	TB-Detecting	Being evaluated by procedures which can identify TB (Evaluations probably for TB)
	Re-Detecting	Re-visiting **TB-Detecting State** after **Interrupted Evaluation**
Treating	First-line treatment	Being treated with first-line TB regimen
	Treatment change	Switching between two TB treatments or temporal treatment interruption due to health conditions.
	Second-line treatment / retreatment	Being treated with second-line TB regimen or any regimen after **Treatment change**
